# Editorial: Biophysical target engagement assays in chemical biology and pharmacological research

**DOI:** 10.3389/fcell.2023.1163966

**Published:** 2023-02-24

**Authors:** Bolormaa Baljinnyam, Nathan P. Coussens, Anton Simeonov

**Affiliations:** ^1^ National Center for Advancing Translational Sciences, National Institutes of Health, Rockville, MD, United States; ^2^ Molecular Pharmacology Laboratories, Applied and Developmental Research Directorate, Frederick National Laboratory for Cancer Research, Frederick, MD, United States

**Keywords:** biophysical methods, target engagement, target deconvolution, mechanism of action, high-throughput screening, pharmacology, differential scanning fluorimetry, cellular thermal shift assay

The application of biophysical methods to pharmacological studies of small molecules or biologicals can confirm target engagement and elucidate the mechanism of action for prospective therapeutics. During the early stages of drug discovery, such information can have major impacts on the overall success of a project by focusing time and resources on the most promising chemical matter and avoiding artifacts (Coussens et al., 2021). A notable example of the later is PTC124 (ataluren) that was identified from a high-throughput screen for compounds that promote the readthrough of non-sense codons in a firefly luciferase (FLuc) reporter gene (Welch et al., 2007). Subsequent biophysical studies demonstrated that PTC124 binds FLuc with a high affinity to both inhibit and stabilize the enzyme with a potency that correlated with the cell-based non-sense codon FLuc reporter assay (Auld et al., 2009; Auld et al., 2010). In addition to target deconvolution and validation, biophysical methods are often implemented to establish structure-activity relationships and support the optimization of chemical leads.

A number of recent technological and methodological advancements have been made in biophysical approaches that can be applied to monitor and measure target engagement in both cell-free and living systems. This special Research Topic of *Frontiers in Cell and Developmental Biology* highlights examples in four peer-reviewed articles, including two original research articles, one review, and one methods article. Collectively these articles describe advancements that can be readily adopted to interrogate the engagement of potential therapeutic targets, from purified recombinant proteins to unlabeled native proteins within intact cells.

Differential scanning fluorimetry (DSF) is a widely used technique to assess the potential of small molecules to directly engage a purified target *in vitro* due to its sensitivity over a wide-range of affinities and amenability to high-throughput workflows. In DSF, ligand binding is typically characterized by a change in the protein melting temperature measured by the binding of an extrinsic environment-sensing dye to hydrophobic patches of a protein as it unfolds in a thermal gradient (Baljinnyam et al., 2020). Because the signal is not specific to the target of interest, artifacts and interferences can be caused by buffer components, detergents, cofactors, binding partners, and protein contaminants. In this special Research Topic, Ronzetti et al. described methods to apply an NTA-complexed red-shifted fluorophore to specifically probe the temperature-induced structural changes in a polyhistidine-labeled target of interest (Ronzetti et al.). The optimized assay was successfully miniaturized to a 1536-well format and validated with a library of 872 compounds against the High-temperature requirement protease A protein from *Borrelia burgdorferi*.

The binding observed between a ligand and its target within a cell-free system does not always translate to a similar interaction within cells. The cellular thermal shift assay (CETSA) bridges this gap by serving as a label-free method that enables the characterization of target engagement in intact cells or cell lysates. Since the CETSA was first reported in 2013 (Martinez Molina et al., 2013), its methods and applications have continued to grow. In this special Research Topic, Tolvanen provides a current review of the advances in CETSA methods and its applications, including the validation of target engagement, target deconvolution from phenotypic screens, high-throughput screening, and structure-activity relationship studies (Tolvanen). This powerful methodology can be applied to assess thermal shifts from 7,000–8,000 proteins simultaneously in a single sample with the ability to distinguish both on-target and off-target activities.

Biophysical approaches to target engagement can be used to select the most promising hit compounds, in addition to identifying their target. An example is presented in this special Research Topic by Taki et al. To discover new anthelmintics for drug-resistant parasites, a phenotypic high-throughput screen of 14,400 compounds was conducted with whole barber’s pole worms, a pathogenic roundworm. The hit compound UMW-868 was discovered to have anthelmintic activity but required target deconvolution to support further optimization. Using thermal proteome profiling, the coauthors identified and quantified 3,678 proteins as part of a “hit-to-target” workflow that ultimately revealed the orphan protein HCO_011565 as a parasite-specific target of UMW-868.

The family of inhibitor of apoptosis proteins (IAPs) regulate apoptosis and immune signaling and are characterized by the presence of a baculovirus IAP repeat (BIR) domain, which mediates protein-protein interactions. The IAP family members are overexpressed in many hematological and solid cancers and are under investigation as therapeutic targets. Inhibitors targeting the BIR of IAPs are currently being tested in clinical trials as monotherapies and in combination with chemotherapy (Cetraro et al., 2022). To accelerate the progress in modulating these oncology targets, Schwalm et al. reported a toolbox that enables the assessment of BIR domain inhibitor selectivity in cells (Schwalm et al.). This toolbox comprises a cell-based IAP family-wide selectivity screening panel based on NanoBRET (Nano Bioluminescence Resonance Energy Transfer) technology that was applied to characterize the selectivity of multiple BIR domain inhibitors, including clinical candidates.

We wish to thank the contributing authors, the *Frontiers* staff and editors who helped assemble this special Research Topic, as well as the scientific reviewers for their thoughtful evaluations of the manuscripts. We hope that the articles in this special Research Topic inspire and enable translational scientists to apply advanced biophysical target engagement assays and accelerate the development of new therapeutics.
